# Early endovascular intervention for unfavorable remodeling of the thoracic aorta after open surgery for acute DeBakey type I aortic dissection: study protocol for a multicenter, randomized, controlled trial

**DOI:** 10.1186/s13063-023-07548-x

**Published:** 2023-08-05

**Authors:** Yu Liu, Ling Li, Zhenghua Xiao, Liqing Peng, Peng Yang, Chen Lu, Yu Zhang, Haiyue Wang, Jia Hu

**Affiliations:** 1 Department of Cardiovascular Surgery, West China Hospital, Sichuan University, No. 37 Guo Xue Alley, Chengdu, 610041 Sichuan China; 2 Chinese Evidence-Based Medicine Center, West China Hospital, Sichuan University, No. 37 Guo Xue Alley, Chengdu, 610041 Sichuan China; 3 Department of Radiology, West China Hospital, Sichuan University, No. 37 Guo Xue Alley, Chengdu, 610041 Sichuan China; 4 Department of Cardiovascular Surgery, West China Guang’an Hospital, Sichuan University, Guang’an, 638000 Sichuan China

**Keywords:** DeBakey type I aortic dissection, Type A aortic dissection, Total arch replacement, Frozen elephant trunk, Aortic remodeling, Endovascular repair, Randomized controlled trial

## Abstract

**Background:**

Total arch replacement with frozen elephant trunk has been developed with promising results for DeBakey type I aortic dissection. However, several problems, such as continuous perfusion of distal false lumen and unfavorable remodeling of distal aorta postoperatively, can seriously affect the long-term outcome. This trial aims to assess the effects of early minimally invasive endovascular repair on distal aortic remodeling and long-term clinical outcomes in patients with dominant false lumen and residual tears in the descending thoracic aorta after total arch replacement and frozen elephant trunk procedure.

**Methods:**

This is a protocol for a two-arm, parallel, multicenter, randomized controlled trial. A total of 154 eligible patients will be recruited from four hospitals in China and randomized on a 1:1 basis either to the experiment group (endovascular repair in addition to routine antihypertensive therapy) or the control group (routine antihypertensive therapy without early surgical treatment). The primary outcome will be the five-year all-cause mortality. The secondary outcomes will include re-intervention, ischemic symptoms, organ dysfunction, and stent-related adverse events.

**Discussion:**

If early minimally invasive endovascular repair could safely and effectively promote distal aortic remodeling and bring favorable long-term outcomes for patients with dominant false lumen and residual tears in the descending thoracic aorta after total arch replacement and frozen elephant trunk technique, it would improve the treatment strategy for DeBakey type I aortic dissection.

**Trial registration:**

Chinese Clinical Trial Registry, CHiCTR2000030050. Registered on 11 March 2020.

**Supplementary Information:**

The online version contains supplementary material available at 10.1186/s13063-023-07548-x.

## Administrative information

**Table:** 

Title {1}	Early endovascular intervention for unfavorable remodeling of the thoracic aorta after open surgery for acute DeBakey type I aortic dissection: study protocol for a multicenter, randomized, controlled trial
Trial registration {2a and 2b}	Chinese Clinical Trial Registry, ID: ChiCTR2000030050. Registered on 11 March 2020
Protocol version {3}	Version 1.0, dated on 6 October 2019
Funding {4}	1·3·5 project for disciplines of excellence–Clinical Research Incubation Project, West China Hospital, Sichuan University (2019HXFH027)
Author details {5a}	Yu Liu^1†^, Ling Li^2†^, Zhenghua Xiao^1^, Liqing Peng^3^, Peng Yang^1^, Chen Lu^1^, Yu Zhang^1^, Haiyue Wang^1^ and Jia Hu^1, 4*^ ^1^Department of Cardiovascular Surgery, West China Hospital, Sichuan University, No. 37 Guo Xue Alley, Chengdu, Sichuan 610,041, China ^2^Chinese Evidence-based Medicine Center, West China Hospital, Sichuan University, No. 37 Guo Xue Alley, Chengdu, Sichuan 610,041, China ^3^Department of Radiology, West China Hospital, Sichuan University, No. 37 Guo Xue Alley, Chengdu, Sichuan 610,041, China ^4^Department of Cardiovascular Surgery, West China Guang’an Hospital, Sichuan University, Guang’an, Sichuan 638,000, China
Name and contact information for the trial sponsor {5b}	Investigator-initiated trial; Jia Hu (Principal investigator); humanjia@msn.com
Role of sponsor {5c}	Jia Hu all contributed to the design and management of this study

## Introduction

### Background and rationale {6a}

Type A aortic dissection (TAAD), especially DeBakey type I aortic dissection, has emerged as one of the most life-threatening conditions because of its extensive dissection and high risk of rupture [1, 2]. According to the International Registry of Aortic Dissection, even with active medical therapy, up to 57% of patients are still unable to avoid death [3]. Therefore, surgery is the first option to lower the early mortality and disability rates of patients with TAAD. The standard open surgery represented by Sun’s procedure (aortic root management/ascending aorta replacement + total arch replacement + frozen elephant trunk implantation) has recently achieved notable short-term efficacy due to advancements in surgical techniques and cardiopulmonary bypass [4]. And the early mortality falls from 11–36% to 6–8% [4, 5].

Blood pressure control and radiologic follow-up are currently the major postoperative managements for patients recovering from type I aortic dissection [6]. However, most patients with type I aortic dissection still have residual dissection and continuous perfusion of the false lumen (FL) in the downstream aorta, which seriously affect the remodeling of the descending thoracic aorta and abdominal aorta [7, 8]. A recent study has reported that FL thrombosis and aortic remodeling are impeded by postoperative tears in the descending aorta [8]. Furthermore, negative remodeling caused by FL patency is related to impaired late survival and an increased risk of re-intervention [9, 10]. Therefore, excluding the residual intimal tears and promoting the thrombosis of the FL in the descending aorta after open surgery is vital for improving the long-term outcomes of type I aortic dissection.

Thoracic endovascular aortic repair (TEVAR), a minimally invasive approach, has shown remarkable success in treating type B aortic dissection (TBAD) and re-intervention following the frozen elephant trunk procedure [2, 11–13]. And open surgery is thought to convert type I aortic dissection into TBAD [14]. Previous research on TBAD has revealed that the FL area greater than 50% of the aortic cross-sectional area in the tracheal bifurcation plane is significantly correlated with poor survival [15]. Nevertheless, despite similarities between residual type I aortic dissection and primary TBAD, the role of early (< 90 days after onset) TEVAR is still unknown in residual aortic dissection with dominant FL (the FL area greater than 50% of the aortic cross-sectional area at the level below the frozen elephant trunk) and tears in the descending thoracic aorta after frozen elephant trunk procedure.

### Objectives {7}

The main purpose of this multicenter trial is to assess the effects of early TEVAR on distal aortic remodeling and long-term clinical outcome in patients with unfavorable remodeling (dominant FL and residual tears) of the descending thoracic aorta after total arch replacement and frozen elephant trunk procedure. We hypothesize that early intervention will promote remodeling of the distal aorta after open operation and reduce mortality, major complications, and re-intervention.

### Trial design {8}

This two-arm, parallel, superiority, multicenter, prospective, randomized controlled trial will be conducted at four centers in China. A total of 154 eligible participants with unfavorable early remodeling of the descending thoracic aorta after total arch replacement with frozen elephant trunk will be divided randomly into experiment group and control group in a ratio of 1:1.

**Fig. 1:**
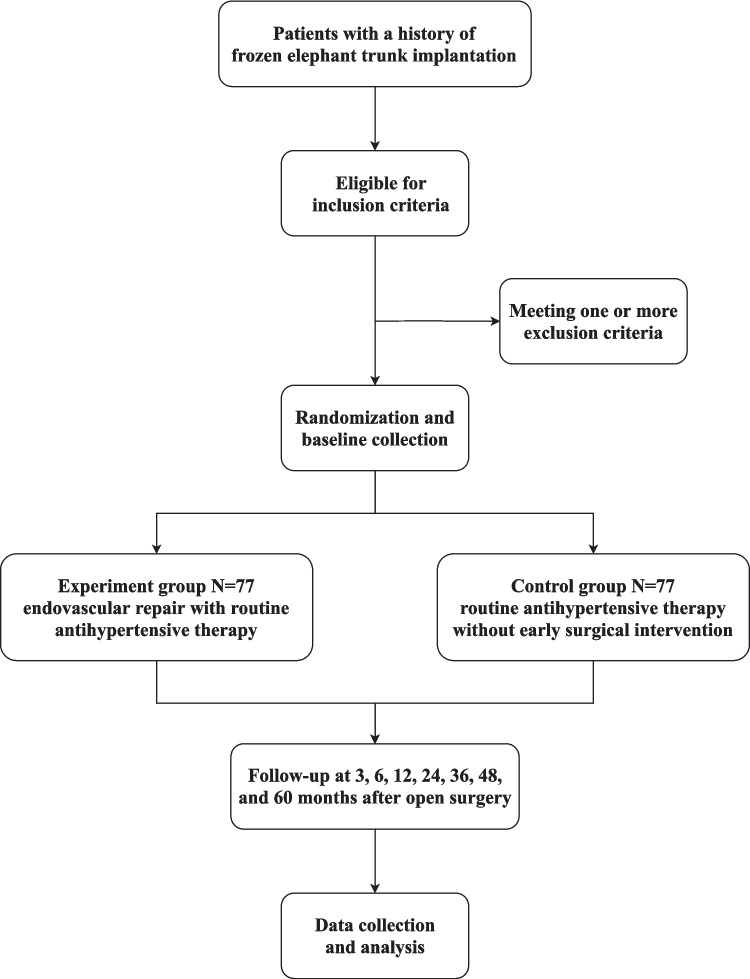
Flowchart of the clinical trial

This protocol is reported according to the Standard Protocol Items: Recommendations for Interventional Trials (SPIRIT) guidelines [16]. The flowchart of the trial is presented in Fig. 1.

## Methods: participants, interventions, and outcomes

### Study setting {9}

Patients will be recruited from four centers in China, including the West China Hospital of Sichuan University, Mianyang Central Hospital, Chengdu Third People’s Hospital, and Mianyang 404 Hospital.

### Eligibility criteria {10}

The 1-month aortic computed tomography angiography (CTA) after open operation will be used to evaluate morphological characteristics, and the inclusion criteria are as follows: (1) patients aged 18 to 80 years old; (2) diagnosed with DeBakey type I aortic dissection within 90 days and 30 days after total arch replacement with frozen elephant trunk procedure; (3) no other aortic surgery history except total arch replacement with frozen elephant trunk implantation; (4) residual tear in the descending thoracic aorta; (5) the area of the FL at the level below the end of the frozen elephant trunk accounts more than 50% of the cross-sectional area of the aorta; (6) revascularization of the left subclavian artery; (7) life expectancy > 12 months; (8) patients can understand the purpose of the trial and voluntarily sign the informed consent form; (9) patients are able to complete follow-up according to the clinical trial.

The exclusion criteria are as follows: (1) pregnant or lactating women; (2) patients who are not suitable for endovascular repair, such as no appropriate vascular approach, allergy to alloy materials and contrast media, and severe renal insufficiency; (3) heritable aortic diseases or inflammatory aortitis.

### Who will take informed consent? {26a}

One fully trained researcher for each center will obtain informed consent from the participants, ensuring that they have a comprehensive understanding of the trial before proceeding.

### Additional consent provisions for collection and use of participant data and biological specimens {26b}

At present, no ancillary studies have been scheduled. However, if future studies require relevant data from participants, additional consent will be obtained accordingly. No biological specimens will be collected for this trial.

## Interventions

### Explanation for the choice of comparators {6b}

Blood pressure control and regular radiologic follow-up are paramount in the postoperative management of patients recovering from type I aortic dissection, which will be implemented in the control group.

### Intervention description {11a}

Participants allocated to the experimental group will be admitted to the hospital promptly for the scheduled endovascular repair which will be performed by surgeons with at least 5 years’ experience. After the standard induction of general anesthesia, the invasive arterial blood pressure of the right radial artery will be monitored. Systemic heparinization (1 mg/kg) will be employed before the establishment of the common femoral artery access via which the endografts will be delivered. During the procedure, the end of the frozen elephant trunk will be used as the proximal landing zone, while the descending aorta above the diaphragm will be chosen as the distal landing zone. Endograft selection and coverage length will be largely left to the discretion of the operating surgeons based on imaging measurements. Patients in the experimental group will concurrently undergo conventional antihypertensive treatment as well.

Participants in the control group will not undergo early surgical treatment but will only receive routine antihypertensive therapy.

Follow-up appointments are scheduled at 3, 6, 12, 24, 36, 48, and 60 months after open surgery. An aortic CTA will be required for every follow-up.

### Criteria for discontinuing or modifying allocated interventions {11b}

The dropout criteria are as follows: (1) the participants request withdrawal; (2) the investigators consider it inappropriate to continue for safety concerns.

### Strategies to improve adherence to interventions {11c}

The participants in the experimental group will undergo surgical intervention promptly upon admission through research channels, which will help to improve adherence.

### Relevant concomitant care permitted or prohibited during the trial {11d}

Relevant concomitant care or intervention will be determined by physicians based on clinical practice guidelines at their discretion during the trial.

### Provisions for post-trial care {30}

Participants will be compensated for any injuries resulting from trial participation in accordance with the relevant laws of China.

### Outcomes {12}

In this trial, the outcomes will be developed according to the standard clinical endpoint of endovascular aortic repair [17, 18]. The primary outcome will be the 5-year all-cause mortality. Secondary outcomes will include re-intervention (indications for re-intervention: aortic diameter ≥ 55 mm, aortic diameter growth rate > 10 mm/year, or pain or malperfusion symptoms [19]), aortic-related mortality, stroke, paraplegia, renal insufficiency, hepatic insufficiency, extremity ischemia, myocardial infarction, endoleak, graft infection, and graft migration. Other morphological outcomes will include segmental aortic remodeling and the FL thrombosis quotient.

The morphological measurements will be analyzed through three-dimensional reconstruction based on CTA images. The celiac trunk will divide the downstream aorta into two segments. Segment A will be from the end of the frozen elephant trunk to the celiac trunk, and segment B will be from the celiac trunk to the aortic bifurcation. The volume of FL, perfused false lumen (PFL), and true lumen (TL) will be measured for the two segments. The FL thrombosis quotient will be calculated using the formula FL_thrombosis quotient_ = 1-PFL/FL and given as a percentage, and the aortic remodeling will be classified into three types based on volumetric changes [20]. Positive aortic remodeling will be defined as a TL increase of > 10% with a stable FL or a FL decrease of > 10% with a stable TL. Changes within 10% will be considered stable, and all other conditions will be regarded as negative remodeling.

### Participant timeline {13}

**Fig. 2:**
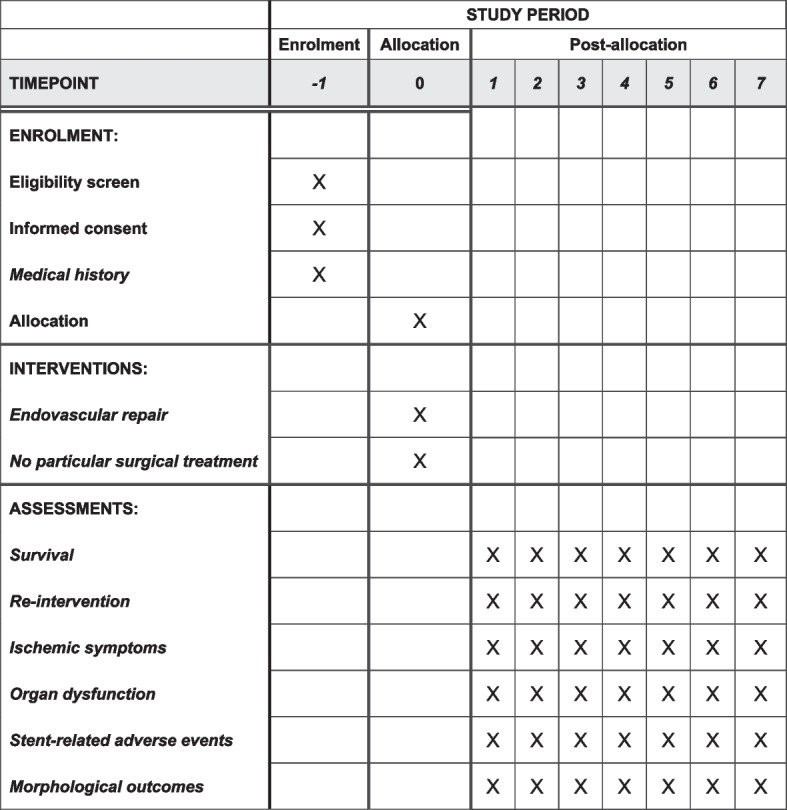
Flowchart of the trial for the schedule of enrolment, interventions and assessments. − 1, > 30 days after the frozen elephant trunk implantation and < 90 days after onset; 0, intervention; 1, 3 months after the frozen elephant trunk implantation; 2, 6 months after the frozen elephant trunk implantation; 3, 12 months after the frozen elephant trunk implantation; 4, 24 months after the frozen elephant trunk implantation; 7, 36 months after the frozen elephant trunk implantation; 8, 48 months after the frozen elephant trunk implantation; 9, 60 months after the frozen elephant trunk implantation

The participant timeline is presented in Fig. 2.

### Sample size {14}

The sample size is calculated based on the 5-year all-cause mortality. According to Roselli’s studies [21, 22], the all-cause mortality is estimated to be 11.0% in the experiment group and 29.8% in the control group. The calculation assumes two-side 5% type I error with 80% power. Therefore, we calculate that we demand 69 participants in each group. Considering the expected dropout rate of 10%, the final sample size is 154 for the entire study.

### Recruitment {15}

Participants in this trial will be recruited from four centers through advertisement and referrals, ensuring a comprehensive approach to reach the desired target sample size.

## Assignment of interventions: allocation

### Sequence generation {16a} and implementation {16c}

The eligible participants will be divided randomly into experiment group and control group in a ratio of 1:1. Randomization will be stratified by centers in block sizes of 2, 4, 6, or 8. And an independent researcher will use SAS 9.3 (SAS Institute Inc., Cary, NC, USA) to generate the randomization sequence, ensuring an unbiased and reliable allocation process.

### Concealment mechanism {16b}

The allocation sequence will be securely stored in sequentially numbered, opaque, sealed envelopes. Once the consent form is completed, the allocation will be revealed to the investigators and participants.

## Assignment of interventions: blinding

### Who will be blinded {17a} and procedure for unblinding if needed {17b}

Given the nature of our intervention, which involves surgical procedures and stent placement, our study is designed as an unblinded trial.

## Data collection and management

### Plans for assessment and collection of outcomes {18a}

Baseline data will be collected after participants have signed informed consent. For the experimental group, intervention-related data will be obtained from the electronic medical records system within 30 days after patient discharge. Follow-up data will be obtained through outpatient visits or telephone interviews after the participants have completed each follow-up aortic CTA examination.

### Plans to promote participant retention and complete follow-up {18b}

Participants will receive transportation allowance for follow-up CTA examinations. Furthermore, a researcher will proactively remind participants of their scheduled CTA follow-up appointments via telephone.

### Data management {19}

Data will be collected in case report forms (CRF), including demographic characteristics, symptoms, signs, laboratory tests, medication, complications, and morphological outcomes. All the CRFs will be filled out independently by one researcher and checked by another researcher. All data collected during the study will be kept for 3 years after the completion of the study.

### Confidentiality {27}

Data access will be strictly limited to the designated study researchers. Any personal information collected during the trial will be treated with utmost confidentiality and handled in strict adherence to the pertinent legal provisions.

### Plans for collection, laboratory evaluation, and storage of biological specimens for genetic or molecular analysis in this trial/future use {33}

Not applicable; no biological specimens will be collected for this trial.

## Statistical methods

### Statistical methods for primary and secondary outcomes {20a}

Baseline characteristics and clinical outcomes will be expressed as numbers (percentages) for categorical variables and mean ± standard deviation for continuous variables. The chi-square test or Fisher’s exact test will be used to analyze categorical variables, and the independent *t* test or the Wilcoxon signed-rank test will be adopted to analyze continuous variables. The Kaplan–Meier method will be performed to generate survival curves, and the log-rank analysis will be used to compare the differences between survival curves. Multivariate Cox regression adjusted by centers will be applied to calculate the hazard ratio risk. *P* < 0.05 will be considered to indicate statistical significance.

### Interim analyses {21b}

There will be no interim analyses in this trial because the primary outcome is the 5-year all-cause mortality which requires long-term follow-up.

### Methods for additional analyses (e.g., subgroup analyses) {20b}

Three prespecified subgroup analyses of age (18–49 vs. 50–80), gender and body mass index (< 24 vs. ≧ 24) will be conducted for the primary outcome by adding an interaction term (e.g., age × group) into the Cox regression model. A sensitivity analysis will be conducted for the primary outcome based on the per-protocol set, which included patients who adhered to the treatment and follow-ups.

### Methods in analysis to handle protocol non-adherence and any statistical methods to handle missing data {20c}

The primary outcome will be analyzed according to the modified intent-to-treat principle, which will minimally exclude patients without receiving the assigned treatment or with missing primary outcome data.

### Plans to give access to the full protocol, participant level-data and statistical code {31c}

Upon reasonable request, the corresponding author will provide access to the full protocol, participant level-data and statistical code.

## Oversight and monitoring

### Composition of the coordinating center and trial steering committee {5d}

The coordinating center for this multi-center clinical trial will be West China Hospital, Sichuan University. We will develop a trial steering committee (TSC) responsible for providing strategic guidance, monitoring the progress of the trial, and making critical decisions related to the trial protocol and overseeing the overall conduct of the study. The TSC will consist of two independent experts in cardiovascular surgery and one independent statistician.

### Composition of the data monitoring committee, its role and reporting structure {21a}

We will also develop a data monitoring committee (DMC) to independently monitor trial data, ensure participant safety, and maintain data integrity and quality throughout the study. The DMC will comprise three independent clinicians, one independent epidemiologist, and one independent statistician. The DMC meeting will be held once a year.

### Adverse event reporting and harms {22}

During the trial, all operations will be performed by surgeons with rich experience in endovascular repair from each center. In the preparation phase, the surgical team has established systematic countermeasures to prevent and deal with adverse events through collaborative discussion with the departments of anesthesiology, vascular surgery, and intensive care medicine, which will help to ensure patient safety. All adverse events will be recorded in the CRF, and serious adverse events will be reported in detail to the Ethics Committee. The investigators will evaluate the relationship between adverse events and interventions.

### Frequency and plans for auditing trial conduct {23}

An independent auditing team will perform formal audit regularly to verify the integrity and reliability of the study data and ensure that the trial is conducted in compliance with ethical standards and protocol.

### Plans for communicating important protocol amendments to relevant parties (e.g., trial participants, ethical committees) {25}

All amendments will undergo in-depth discussions within the TSC before being submitted to the Ethics Committee for approval.

### Dissemination plans {31a}

The research results of this trial will be reported in domestic and international academic conferences, as well as in peer-reviewed journals.

## Discussion

Type I aortic dissection is still a challenge for cardiovascular surgeons. The frozen elephant trunk has achieved promising early and mid-term outcomes with the purpose of closing the intimal tear at the proximal descending aorta and promoting thrombosis of the false lumen along the stent graft [23, 24]. However, residual tears and continuous perfusion of the FL in the downstream aorta adversely affect the long-term prognosis, which will lead to secondary interventions in downstream segments [7, 8]. Several studies have demonstrated the safety and effectiveness of TEVAR in distal aortic re-interventions after frozen elephant trunk implantation [11–13, 25]. However, these studies were all single-center retrospective research, and endovascular re-interventions were mainly used to address distal aortic aneurysmal dilatation. Another single-center study enrolled 26 patients with dissection extending to the aortic bifurcation and compared downstream aortic remodeling following the standard or the elongated (an additional thoracic stent-graft implanted down to the celiac trunk within 30 days) frozen elephant trunk procedure [26]. It showed that an extra stent-graft appeared to improve aortic remodeling and lower the risk of distal aortic re-interventions. Nonetheless, it is important to note that routine extensive descending aortic coverage with a short interval increases the risk of spinal cord ischemia. Therefore, in our study, TEVAR will be performed at least 30 days after the frozen elephant trunk procedure but within 90 days of the onset to promote collateral revascularization of intercostal arteries and reduce the influence of rigid thick intimal flap on the aortic remodeling. Meanwhile, we will only enroll patients with dominant FL and residual tears in the descending thoracic aorta after the frozen elephant trunk implantation because they will be at higher risk for distal aortic adverse events and negative remodeling. Thus, evaluating early intervention is critical for these patients.

However, our study has several limitations. First, the patients and surgeons cannot be blinded to the intervention. However, the primary outcome of our trial is all-cause mortality, which will unlikely be affected by blinding. Second, the sample size is still relatively small, although we have carefully calculated it. Another limitation is that our four centers are all located in southwest China, which will limit the generalizability. Therefore, a nationwide or even worldwide large-scale clinical trial is still warranted in the future.

This trial will evaluate the efficacy of early endovascular repair in promoting remodeling of the downstream aorta and improving clinical prognosis in patients with undesirable early remodeling outcomes after total arch replacement with the frozen elephant trunk technique. We expect this trial will fill the gap in this research field and provide reliable clinical data to perfect the treatment strategy for type I aortic dissection.

### Trial status

The version number of this protocol is 1.0, dated on 6 October 2019. This trial began patient recruitment in June 2020. Completion of recruitment is expected on 31 December 2023.

### Supplementary Information

**Additional file 1.**

## Data Availability

The datasets analyzed during the current study will be available from the corresponding author on reasonable request.

## Abbreviations

TAAD: Type A aortic dissection
FL: False lumen
TEVAR: Thoracic endovascular aortic repair
TBAD: Type B aortic dissection
SPIRIT: Standard Protocol Items: Recommendation for Interventional Trials
CTA: Computed tomography angiography
PFL: Perfused false lumen
TL: True lumen
CRF: Case report form
TSC: Trial steering committee
DMC: Data monitoring committee
