# Supplementary material for: Early endovascular intervention for unfavorable remodeling of the thoracic aorta after open surgery for acute DeBakey type I aortic dissection: study protocol for a multicenter, randomized, controlled trial
Source: Trials. 2023 Aug 5;24:496. doi: 10.1186/s13063-023-07548-x (PMC10403865; doi:10.1186/s13063-023-07548-x)
Supplement: Supplementary file 1 — Additional file 1. [file 13063_2023_7548_MOESM1_ESM.docx]

**Version:** 3.0 **Version Date:** March 4, 2020

# Informed Consent Form

Dear patient,

We invite you to participate in a research titled "Early endovascular intervention for unfavourable remodeling of the thoracic aorta after open surgery for acute Debakey type Ⅰ aortic dissection: a multicenter, randomized, controlled trial" which has been funded by the 1·3·5 project for disciplines of excellence–Clinical Research Incubation Project, West China Hospital, Sichuan University (2019HXFH027). This trial will be conducted at West China Hospital of Sichuan University, Mianyang Central Hospital, Chengdu Third People's Hospital, and Mianyang 404 Hospital. It is estimated that there will be 154 voluntary participants in this trial. Ethical approval has been obtained from the Biomedical Ethics Committee of West China Hospital (ref approval no. 2019[1028])

1. **Why is this study being conducted?**

Type A aortic dissection, particularly Debakey type I, is a life-threatening condition characterized by extensive dissection and a high risk of rupture. Surgery represented by Sun's procedure (aortic root management/ascending aorta replacement + total arch replacement + frozen elephant trunk implantation) is the preferred option to reduce early mortality and disability rates. Blood pressure control and radiologic follow-up are currently the major postoperative managements for patients recovering from type I aortic dissection. However, many patients still have residual dissection and continuous perfusion of the false lumen, which hinders the remodeling of the downstream aorta. The main purpose of this multicenter trial is to assess the effects of early endovascular repair on distal aortic remodeling and long-term clinical outcome in patients with unfavourable remodeling (dominant false lumen and residual tears) of the descending thoracic aorta after total arch replacement and frozen elephant trunk procedure.

1. **What do you need to do?**

Participants will be randomly assigned to either the experimental group or the control group in a 1:1 ratio. In the experimental group, participants will undergo endovascular repair along with routine antihypertensive therapy, while participants in the control group will receive only conventional antihypertensive treatment without early surgical intervention. Follow-up appointments will be scheduled at 3, 6, 12, 24, 36, 48, and 60 months after the open surgery. An aortic computed tomography angiography will be required for each follow-up session.

**3. What treatment options are available?**

Blood pressure control and radiologic follow-up are currently the primary postoperative management strategies for patients in recovery from type I aortic dissection. In this trial, participants will be randomly assigned to either undergo early endovascular repair in addition to routine antihypertensive therapy, or receive only conventional antihypertensive treatment without early surgical intervention. Non-participants will receive routine antihypertensive therapy, as there are no alternative options available.

**4. Who is eligible to participate, and who is not?**

The inclusion criteria are as follows: (1) patients aged 18 to 80 years old; (2) diagnosed with Debakey type I aortic dissection within 90 days and 30 days after total arch replacement with frozen elephant trunk procedure; (3) no other aortic surgery history except total arch replacement with frozen elephant trunk implantation; (4) residual tear in the descending thoracic aorta; (5) the area of the false lumen at the level below the end of the frozen elephant trunk accounts more than 50% of the cross-sectional area of the aorta; (6) revascularization of the left subclavian artery; (7) life expectancy > 12 months; (8) patients can understand the purpose of the trial and voluntarily sign the informed consent form; (9) patients are able to complete follow-up according to the the clinical trial.

The exclusion criteria are as follows: (1) pregnant or lactating women; (2) patients who are not suitable for endovascular repair, such as no appropriate vascular approach, allergy to alloy materials and contrast media, and severe renal insufficiency; (3) heritable aortic diseases or inflammatory aortitis.

Morphological characteristics will be evaluated according to the one-month aortic computed tomography angiography after the open operation.

**5. What are the risks associated with participating in the study?**

There is no significant difference between the endovascular procedures for residual dissection after Debakey type I aortic dissection and type B aortic dissections. Potential complications for endovascular procedure include endoleak, vascular injury, rupture, stroke, Spinal cord ischemia, stent-graft infection, stent graft-induced new entry tear, renal dysfunction, and misplacement of the stent-graft into the false lumen. There are no additional risks compared to conventional endovascular surgery.

1. **What are the potential benefits of participating in the study?**

By participating in this study, there is a possibility of improvement in your medical condition. Additionally, this study can help determine which treatment approach can be safer and more effective in treating other patients with similar conditions as yours.

1. **Is there a cost associated with participating in the study?**

This study ensures fair and equitable selection of participants, and no fees are imposed on individuals who choose to participate. All participants voluntarily sign an informed consent form before enrolling in the trial. During the hospitalization period, participants are responsible for covering the costs associated with their treatment. Additionally, participants will receive transportation allowance for follow-up CTA examinations. Participants will be compensated for any injuries resulting from trial participation in accordance with the relevant laws of China.

1. **Is personal information treated as confidential?**

The research data of the participants will be stored at West China Hospital, Sichuan University. The researchers, regulatory authorities, and ethics review committee will have access to the participants' medical records for the purpose of the study. Any public reports or publications regarding the study results will not disclose the personal identities of the participants. We will make every effort to protect the privacy and personal information of the participants' medical data within the limits allowed by law.

1. **Is participation in the study mandatory?**

Participation in this study is completely voluntary. You have the right to refuse participation or withdraw from the study at any stage without facing any discrimination or retaliation. Your medical treatment and rights will not be affected by your decision. If you choose to withdraw from the study, please contact your doctor to ensure appropriate management of your condition.

**Participant Declaration:** I have read the above description of the study, and my research personnel have provided me with a comprehensive explanation of the purpose, procedures, potential risks, and potential benefits of participating in this study. They have also addressed all my questions regarding the study. I voluntarily agree to participate in this research.

**Participant：**

Name： Signature：

Date： 　　Tel：

Legal representative if the participant is unable to give permission:

Name： Signature：

Date： 　　Tel：

Reason：

**Researcher's Statement:** I have provided the volunteer mentioned above with a detailed explanation of the relevant details of participating in this study and have provided them with an original copy of the informed consent form that they have signed. I confirm that I have explained the nature of the study to the participant, including the potential risks and benefits, free participation or compensation, harm and indemnification, voluntary participation, and confidentiality, in accordance with ethical principles and requirements.

**Researcher：**

Name： Signature：

Date： 　　Tel：

**The Biomedical Ethics Committee of West China Hospital**  **Tel: (+86) 028-85422654**
